# Hosoya entropy analysis of some fullerene structures

**DOI:** 10.1186/s11671-025-04255-1

**Published:** 2025-07-01

**Authors:** Ali N. A. Koam, Muhammad Faisal Nadeem, Ali Ahmad, Abdullah Ali H. Ahmadini, Bahreselam Sielu Abraha, Ibtisam Masmali

**Affiliations:** 1 Department of Mathematics, College of Science, Jazan University, P.O. Box. 114, 45142 Jazan, Kingdom of Saudi Arabia; 2 Department of Mathematics, COMSATS University Islamabad, Lahore Campus, Lahore, 54000 Pakistan; 3 Department of Computer Science, College of Engineering and Computer Science, Jazan University, Jazan, Kingdom of Saudi Arabia; 4 Mainefhi College of Engineering and Technology, Mainefhi, Asmara, Eritrea

**Keywords:** Carbon allotropes, Fullerene stability, Fullerene structures, Hosoya entropy, Molecular complexity, Nanotechnology

## Abstract

This study examines the structural complexity of fullerene graphs using Hosoya entropy as a measure. The entropy values were calculated for various fullerene structures, including $F^s_{3,1},$ $F^s_{4,2}$ and fullerenes ranging from C20 to C100.The relationship between the size of the fullerenes and the entropy is intuitively clear: the larger the fullerenes, the higher the value of entropy because of increased structural complexity and diversity of equivalence classes. Smaller fullerenes, like C20, have lower entropy, a consequence of their simpler and more symmetrical molecular structure. These findings provide theoretical insights into structural intricacies of fullerenes and their possible applications in material science and nanotechnology.

## Introduction

Fullerene graphs constitute a unique class of allotropes of carbon with molecular geometries resembling architect Buckminster Fuller’s geodesic domes [1]. The molecules exhibit various symmetrical shapes, such as spherical [2], elliptical [3], and cylindrical shapes [4]. Buckminsterfullerene ($C_{60}$) with a structure resembling a soccer ball-shaped truncated icosahedron [5] is especially renowned. Exceptional chemical stability [6] and mechanical properties [7] in fullerenes render them useful in high-tech nanomaterials in protective coatings, lubricants, and nanotubes. Furthermore, biomedical applications of fullerenes are very significant, especially as drug delivery agents to encapsulate therapeutic molecules for targeted therapy, especially in cancer therapy [8–10]. Furthermore, because of their intrinsic antioxidant activity, fullerenes have great potential in therapeutic treatments against diseases due to oxidative stress.

As a consequence, graph entropy can take into account such a broad set of graph features as vertex degrees, an edge distribution, or even other graph features. The more complex a structure would be, or more accurately, the more random it would be, with a rising graph entropy, and vice versa, with a decrease in entropy, it would mean something simpler or more periodic. Graph entropy can be utilized in a variety of applications, from network analysis to machine learning and optimisation, in which it helps determine structure and relationships in vast amounts of data.

Graph measures based on Shannon entropy have been amply studied. Among the first of them was the one proposed by Rashevsky [11] and afterwards developed by Mowshowitz and Dehmer [12]. This information content measure tries to quantify the complexity of the graph structure. Shannon entropy was derived from a probability distribution produced from the symmetries of the graph by the developed procedure [13]. For example, the automorphism group of a graph partitions its vertices into orbits, for which there exists a probability distribution depending on the size of each orbit divided by the overall number of vertices. Shannon entropy is subsequently used on this distribution, resulting in the graph’s information content. These entropy-based measures have values in the study of problems emerging from mathematical chemistry, computational physics, and pattern recognition. Since then, graph entropy has held an important place in network science as the means for quantitative analysis of networked structures in all possible disciplines, with a considerable number of studies dedicated to exploring its use.

The Hosoya polynomial which is also a counting polynomial introduced by Hosoya in 1988, used to count the distance between vertices having different lengths [14]. Then in 1993, Gutman describes that most of the distance-based graph invariants can be found from Hosoya polynomial [15]. When two vertices *u* and *v* have the same partial Hosoya polynomial, or when both *u* and *v* have the same number of vertices at distance *k* for all *k* from 1 to the maximum distance of *G*, they are known as Hosoya-equivalent.

$$H(G,x)=\sum _{u,v\in E(G)} x^{d(u,v)}.$$

Two vertices, $u$ and $v$, are considered Hosoya-equivalent, or $H$-equivalent for short, when their sequence values $s_i(u)$ and $s_i(v)$ match for all $1 \le i \le \rho (G)$, where $\rho (G)$ is the vertex partition function. The collection of $H$-equivalent vertices forms a partition of the graph’s vertex set. This partition is known as the $H$-partition, comprising distinct $H$-equivalence classes. Let $H_1, H_2, \ldots , H_h$ denote these $H$-equivalence classes within the graph $G$.

The Hosoya entropy (also referred to as $H$-entropy) of $G$ is defined by the following equation:

1 $$\begin{aligned} H(G) = - \sum _{i=1}^{h} \frac{|H_i|}{|V|} \log \left( \frac{|H_i|}{|V|} \right) \end{aligned}$$

where $|H_i|$ represents the number of vertices in each equivalence class, and $|V|$ is the total number of vertices in the graph $G$.

A few studies recently have engaged in the computation and analysis of entropy in various molecular and network structures, focusing on the impact of entropy measures concerning their applications in these diverse domains. Song et al. explored hex-derived networks, which are those formed from hexagonal shapes-a common form observed in many aspects of chemistry and materials science [16]. Liu et al. [17] have furthered this work by underpinning the study in a well-known material in catalysis and adsorption processes-zeolite ACO. Zhao et al. [18] advanced this field of study by researching the entropy of dominating David-derived networks, applied in communication, and hence computer networks. The authors developed a framework necessary for the calculation and analysis of entropy in these networks, where the statistics then in turn controlled the behavior of each type of network. The present study underlines the role of entropy as a proper tool for testing efficiency and robustness of network structures and, therefore, its applicability in optimization processes in network design and functionality. In a more recent work, the authors Zhao et al. [19] focused their attention on superlattices, a structure important in electronics and material design. In this direction, the reduced reverse degree-based topological indices of graphyne and graphdiyne nanoribbons by Zaman et al. [20] are important contributions in chemical analysis. Irfan et al. [21] extend the contribution to topological indices through their work on M-polynomial derivation for line graphs of chain silicate networks and H-naphtalenic nanotubes. These indices provide further insight into the structural and bonding intricacies of silicates and nanotubes that are helpful in developing nanotechnology. Al Khabyah [22] discusses the mathematical properties and topological indices of two different chemical networks. The present work emphasizes the role that topological indices play in enhancing the understanding of complex chemical structures, with a focus on algebraic and graphical properties. Lal et al. discuss the topological properties of different structures [23, 24]. Ahmad et al. [25] contribute to this by providing closed-form expressions for the Omega and Sadhana polynomials of nanosheets, as shown by the analytics, which furthers the design and analysis of nanomaterials. Alali et al. [26] consider the structure of graphs over the commutative ring $\mathbb {Z}_m$ and investigate the topological indices and entropies of this with the help of M-polynomial. Given graph theory and, particularly, chemical networks, Alamer et al. [27] consider cacti graphs completely determining the minimum bounds for Zagreb eccentricity indices. This contributes to optimization and analysis in molecular graphs important for manifold chemical studies. The application of QSPR to Alzheimer’s compounds with topological indices and regression models by Sardar and Hakami [28] shows the relevance of topological approaches in pharmaceutical chemistry and drug design.

The understanding of the molecular structure of fullerenes’ complexity is impossible without a theoretical analysis. In this work, a graph-based measure for the molecular complexity-Hosoya entropy-is applied to fullerenes. While previous fullerene research typically considers aspects of isomer diversity and molecular symmetry from an experimental viewpoint, the approach followed here is strictly theoretical, concentrating on mathematical quantification of the phenomenon. Hosoya entropy represents a further contribution toward classical topological indices in the light of its capability to account for the distribution of equivalence classes, hence representing a novel point of view with respect to symmetry and structural diversity.

## Methodology and results

**Fig. 1:**
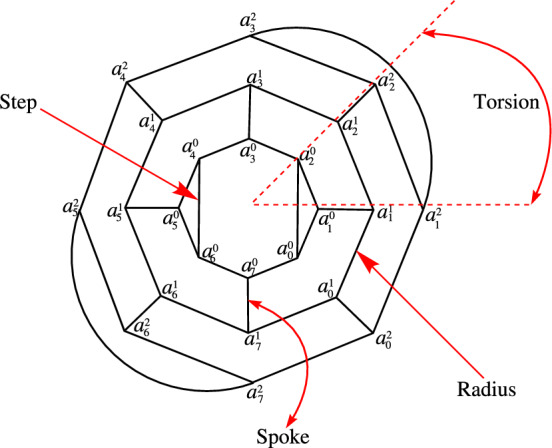
(3,6) Fullerene

In [29], Fowler et al. studied the structure of 3-connected (3,6)-fullerene graphs and gave the triple parameter (*r*, *s*, *t*) characterization for these graphs. Those parameters should fulfill: $r \ge 1$, $s \ge 4$ where even number, $0 \le t < s$ and $t \equiv r$ mod 2. In the terminology, *r* is the radius or number of rings; *s* is the number of spokes in each layer, which is twice the number of steps; and *t* is the twist or torsion. Note that different choices for these parameters can sometimes give the same graph. We define the vertex set $V({F}_{r,t}^{s})$ and the edge set $E({F}_{r,t}^{s})$ of ${F}_{r,t}^{s}$ as follow:

$$V({F}_{r,t}^{s})=\{a_{i}^{j}: i=0,1,2,\ldots ,2s-1, j=0,1,2\ldots ,r-1\},$$

$$E({F}_{r,t}^{s})=A\cup B\cup C \cup S \cup T,$$

where

$$A=\{a_{i}^{j}a_{i+1}^{j}: i=0,1,\ldots ,2s-1, j=0,1\ldots ,r-1\},$$

$$B=\left\{ a_{i}^{j}a_{i}^{j+1}:i=1,3,\ldots ,2\,s-1,j\in \left\{ 0,1,\ldots ,r-2\right\} \text { is even}\right\} ,$$

$$C=\left\{ a_{i}^{j}a_{i}^{j+1}:i=0,2,\ldots ,2\,s-2,j\in \left\{ 0,1,\ldots ,r-2\right\} \text { is odd}\right\} ,$$

$$S=\{a_{2i}^{0}a_{2s-2(i-1)}^{0}: i=1,2,,\ldots ,\frac{s}{2}\},$$

$$T=\left\{ a_{t-i}^{r-1}a_{t+2+i}^{r-1}:i=0,2,\ldots ,2s-2\right\} ,$$

subscripts are taken modulo 2*s*. For illustration, see Fig. 1.

### Hosoya entropy of fullerene $F^s_{3,1}$

First we calculate the Hosoya entropy of fullerenes $F^s_{3,1}$ for $r=3$, $t=1$ and $s\ge 4(even)$. For this we partitioned the graph into H-classes which will be further used to compute the Hosoya entropy of that particular graph.

For $F^4_{3,1}$, the vertices are partitioned into five equivalence classes: four classes contain 4 vertices each, and one class contains 8 vertices. Using this partitioning, the proportions are calculated as $\lambda _1 = \frac{1}{6}$ for the first four classes and $\lambda _2 = \frac{1}{3}$ for the fifth class. Substituting these into the entropy formula gives $H(F^4_{3,1}) = \ln (3) + \frac{2}{3} \ln (2)$. A similar process is followed for different fullerenes, see Table 1.

**Table 1:** Hosoya Entropy for $F_{3,1}^{s}$ Fullerenes

Fullerene	Vertex count	Number of classes	Cardinality of classes	Hosoya entropy
$F_{3,1}^4$	24	5	4, 4, 4, 4, 8	$\ln (3) + \frac{2}{3} \ln (2)$
$F_{3,1}^6$	36	8	4 (× 7), 8	$\frac{10 \ln (3)}{9} + \frac{2 \ln (2)}{9}$
$F_{3,1}^8$	48	12	4 (× 12)	$\ln (12)$
$F_{3,1}^{10}$	60	14	4 (× 13), 8	$\ln (15) - \frac{2}{15} \ln (2)$
$F_{3,1}^{14}$	84	23	2(× 4), 4(× 19)	$\frac{21}{9} \ln (15) - \frac{1}{9} \ln (2)$
$F_{3,1}^{18}$	108	6	12(× 3), 24(× 3)	$\ln (9) - \frac{2}{3} \ln (2)$
$F_{3,1}^{22}$	132	32	4(× 31), 8	$\ln (33) - \frac{2}{33} \ln (2)$
$F_{3,1}^{24}$	144	35	4(× 34), 8	$\ln (36) - \frac{2}{36} \ln (2)$
$F_{3,1}^{26}$	156	38	4(× 37), 8	$\ln (39) - \frac{2}{39} \ln (2)$
$F_{3,1}^{28}$	168	41	4(× 40), 8	$\ln (42) - \frac{2}{42} \ln (2)$

#### Hosoya entropy of fullerene $F^s_{3,1}$ for $s\ge 30$

 For fullerenes $F^s_{3,1}$, where $s \ge 30$ and $s$ is an even number, the graph is divided into multiple Hosoya equivalence classes. Specifically, the first class, $H_1$, contains 8 vertices, and the remaining classes, $H_2$ to $H_{\frac{3\,s-4}{2}}$, each contain 4 vertices.

The general expression for the Hosoya entropy of these fullerenes is given by:

$$\begin{aligned} H(F^s_{3,1}) = \frac{3 s \ln \! \left( \frac{1}{3 s -4}\right) +\ln \! \left( 2\right) \left( 3 s +4\right) }{3 s -4} \end{aligned}$$

This formula applies for any even value of $s \ge 30$, providing a concise way to compute the entropy for larger fullerene graphs in this series.

### Hosoya entropy of fullerene $F^s_{4,2}$

The vertex and edge sets of the graph $F^s_{4,2}$, for $k\ge 1$, are defined as follows.

$$\begin{aligned} V(F^s_{4,2})=&\left\{ u,v\right\} \cup \{u_{i}^{j},v_{i}^{j}: i=0,1,2; \ j=0,1,\ldots ,s\},\\ E(F^s_{4,2})=&\left\{ uu_{i}^{0}, vv_{i}^{0}: i=0,1,2\right\} \cup \left\{ u_{i}^{j}v_{i}^{j}: i=0,1,2; \ j=1,2,\ldots ,s\right\} \\  &\cup \left\{ u_{i}^{s}v_{i}^{0},u_{i}^{s}v_{i-1}^{0}: i=0,1,2 \right\} \\&\cup \left\{ u_{i}^{j}v_{i}^{j+1},u_{i}^{j}v_{i-1}^{j+1}: i=0,1,2\text { and }j\text { is even, }0\le j\le s-1\right\} \\&\cup \left\{ u_{i}^{j}v_{i}^{j+1},u_{i}^{j}v_{i+1}^{j+1}:i=0,1,2\text { and }j\text { is odd, }1\le j\le s-1 \right\} , \end{aligned}$$

**Fig. 2:**
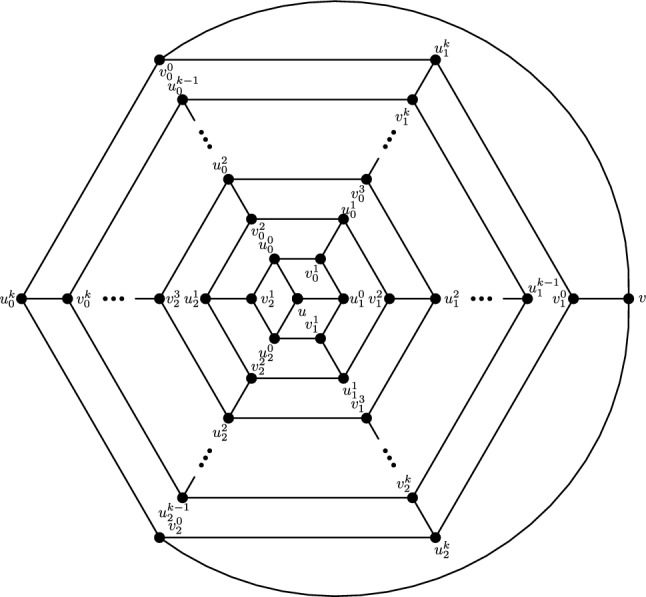
(4,6) Fullerene

For illustration, see Fig. 2.

#### Hosoya entropy of fullerene $F^4_{4,2}$

 For Hosoya entropy of different classes of fullerene $F^s_{4,2}$, where $s=4, 6, 8, 10, 12, 14, 16, 18$ see Table 2.

**Table 2:** Hosoya Entropy for $F_{4,2}^{s}$ Fullerenes

Fullerene	Vertex count	Number of classes	Cardinality of classes	Hosoya entropy
$F_{4,2}^4$	32	6	4(× 4), 8, 8	$3\ln (2)$
$F_{4,2}^6$	48	11	4(× 10), 8	$\ln (6) + \frac{5}{6} \ln (2)$
$F_{4,2}^8$	64	14	4 (x14), 8	$-\frac{31 \ln \! \left( 2\right) }{8}$
$F_{4,2}^{10}$	80	20	4 (× 20)	$\ln (20)$
$F_{4,2}^{12}$	96	24	4(× 24)	$\ln (24)$
$F_{4,2}^{14}$	112	27	4(× 26), 8	$\frac{26}{28} \ln (28) + \frac{1}{14} \ln (14)$
$F_{4,1}^{16}$	128	31	4(× 30), 8	$\frac{79 \ln \! \left( 2\right) }{16}$
$F_{4,1}^{18}$	136	33	4(× 32), 8	$\frac{64 \ln \! \left( 34\right) }{69}+\frac{\ln \! \left( 17\right) }{17}$

#### General Hosoya entropy of fullerene $F^s_{4,2}$

 For fullerene $F^s_{4,2}$, where $s \ge 20$ and $s$ is even, the graph is divided into equivalence classes, with the first class $H_1$ containing 8 vertices and the remaining classes $H_2$ to $H_{2\,s-2}$ each containing 4 vertices.

The general expression for the Hosoya entropy is:

$$\begin{aligned} H(F^s_{4,2}) = \frac{\left( s-1\right) \ln \! \left( 2\right) +s \ln \! \left( s\right) }{s} \end{aligned}$$

This formula provides the entropy for any even $s \ge 20.$

## Some other classes of fullerene

### Hosoya entropy of fullerene $H(C_{20})$

**Fig. 3:**
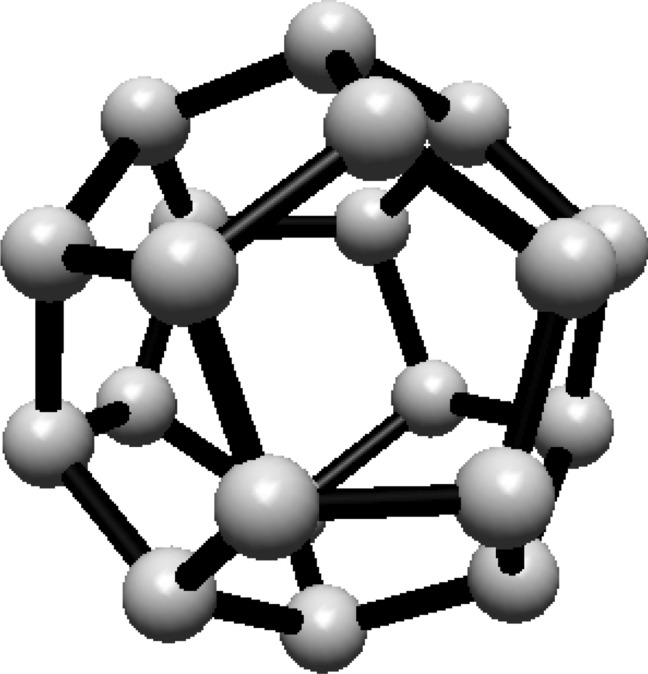
$$ C_{20} $$ Fullerene Structure

For the fullerene $C_{20}$ see Fig. 3, the graph consists of one Hosoya equivalence class, $H_1$, containing all 20 vertices.

Given that all vertices belong to one class, the normalized proportion is $\lambda _1 = 1$. Substituting this into Eq. (1):

$$\begin{aligned} H(C_{20}) = 1 \cdot \ln (1) = 0 \end{aligned}$$

Thus, the Hosoya entropy of $C_{20}$ is $H(C_{20}) = 0$.

### Hosoya entropy of fullerene $H(C_{24})$

**Fig. 4:**
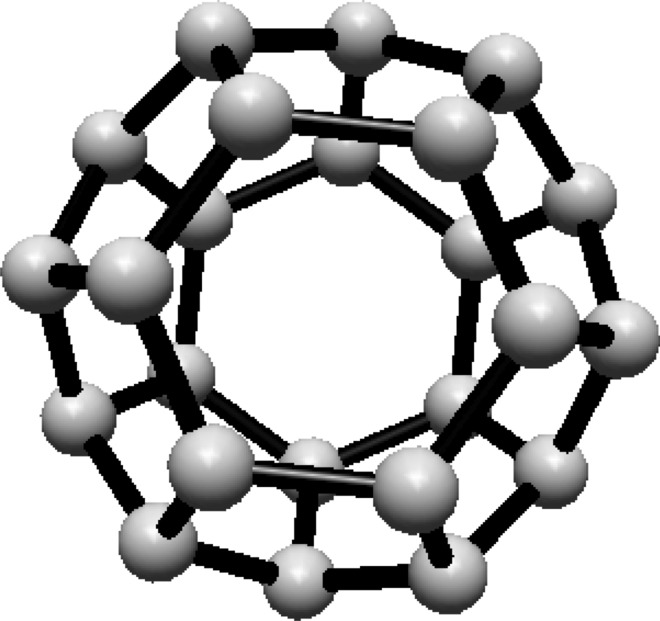
$$ C_{24} $$ Fullerene Structure

For the fullerene $C_{24}$ see Fig. 4, the graph is divided into two Hosoya equivalence classes, with $H_1$ and $H_2$ containing 12 vertices each.

The normalized proportions for the two classes are $\lambda _1 = \lambda _2 = \frac{1}{2}$. Substituting this into Eq. (1):

$$\begin{aligned} H(C_{24}) = -\ln \left( \frac{1}{2}\right) \end{aligned}$$

This simplifies to:

$$\begin{aligned} H(C_{24}) = \ln (2) \end{aligned}$$

Thus, the Hosoya entropy of $C_{24}$ is $H(C_{24}) = \ln (2)$.

### Hosoya entropy of fullerene $H(C_{26})$

**Fig. 5:**
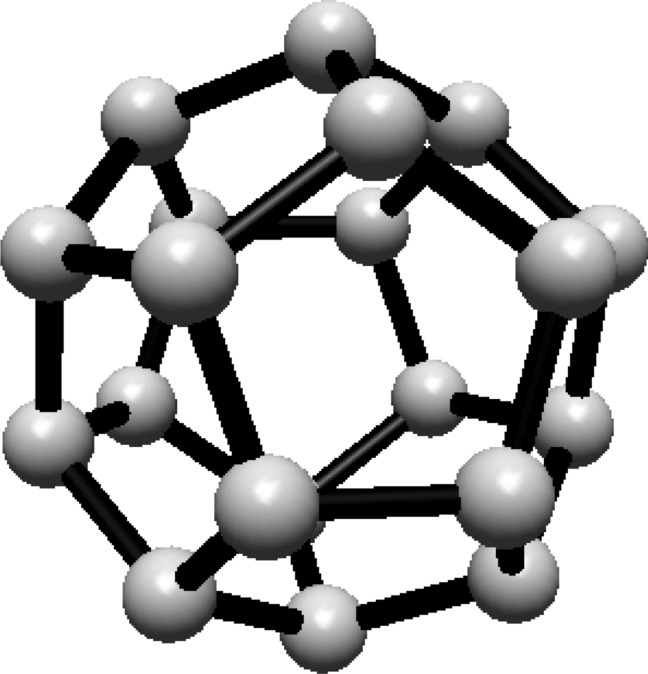
$$ C_{26} $$ Fullerene Structure

For the fullerene $C_{26}$ see Fig. 5, the graph is divided into two Hosoya equivalence classes, with $H_1$ containing 8 vertices and $H_2$ containing 18 vertices.

The normalized proportions are $\lambda _1 = \frac{4}{13}$ and $\lambda _2 = \frac{9}{13}$. Substituting these into Eq. (1):

$$\begin{aligned} H(C_{26})  &   = -\frac{4}{13} \ln \left( \frac{4}{13}\right) \\  &   \quad - \frac{9}{13} \ln \left( \frac{9}{13}\right) \end{aligned}$$

Breaking this down:

$$\begin{aligned} H(C_{26}) = -\frac{4}{13} \ln (4) - \frac{9}{13} \ln (9) + \ln (13) \end{aligned}$$

Thus, the Hosoya entropy of $C_{26}$ is:

$$\begin{aligned} H(C_{26}) = \ln (13) - \frac{4}{13} \ln (4) - \frac{9}{13} \ln (9) \end{aligned}$$

### Hosoya entropy of fullerene $H(C_{28})$

**Fig. 6:**
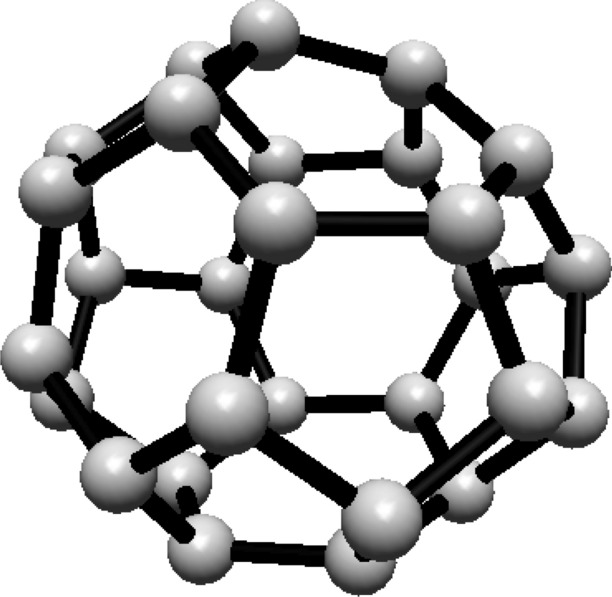
$$ C_{28} $$ Fullerene Structure

For the fullerene $C_{28}$ see Fig. 6, the graph is divided into two Hosoya equivalence classes, with $H_1$ containing 24 vertices and $H_2$ containing 4 vertices.

The normalized proportions are $\lambda _1 = \frac{1}{7}$ and $\lambda _2 = \frac{6}{7}$. Substituting these into Eq. (1):

$$\begin{aligned} H(C_{28})  &   = -\frac{1}{7} \ln \left( \frac{1}{7}\right) \\  &   \quad - \frac{6}{7} \ln \left( \frac{6}{7}\right) \end{aligned}$$

Simplifying:

$$\begin{aligned} H(C_{28}) = \ln (7) - \frac{6}{7} \ln (6) \end{aligned}$$

Thus, the Hosoya entropy of $C_{28}$ is:

$$\begin{aligned} H(C_{28}) = \ln (7) - \frac{6}{7} \ln (6) \end{aligned}$$

#### Hosoya entropy of fullerene $H(C_{30})$

**Fig. 7:**
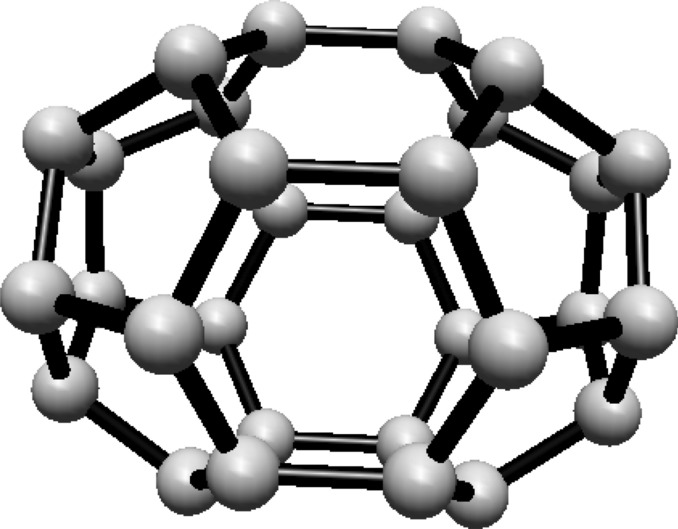
$$ C_{30} $$ Fullerene Structure

 For the fullerene $C_{30}$ see Fig. 7, the graph consists of five Hosoya equivalence classes. The cardinalities of these classes are 4, 5, 5, 6, and 10, respectively.

The normalized proportions are $\lambda _1 = \frac{2}{15}$, $\lambda _2 = \frac{1}{6}$, $\lambda _3 = \frac{1}{5}$, and $\lambda _4 = \frac{1}{3}$. Substituting these into Eq. (1):

$$\begin{aligned} H(C_{30})  &   = -\frac{2}{15} \ln \left( \frac{2}{15}\right) - \frac{1}{6} \ln \left( \frac{1}{6}\right) \\  &   \quad - \frac{1}{5} \ln \left( \frac{1}{5}\right) - \frac{1}{3} \ln \left( \frac{1}{3}\right) \end{aligned}$$

Simplifying:

$$\begin{aligned} H(C_{30})  &   = \frac{2}{15} \ln (15) - \frac{2}{15} \ln (2) + \frac{1}{3} \ln (6) \\  &   \quad + \frac{1}{5} \ln (5) + \frac{1}{3} \ln (3) \end{aligned}$$

Thus, the Hosoya entropy of $C_{30}$ is:

$$\begin{aligned} H(C_{30}) = \frac{1}{3} \ln (5) - \frac{4}{5} \ln (3) + \frac{1}{5} \ln (5) \end{aligned}$$

#### Hosoya entropy of fullerene $H(C_{32})$

**Fig. 8:**
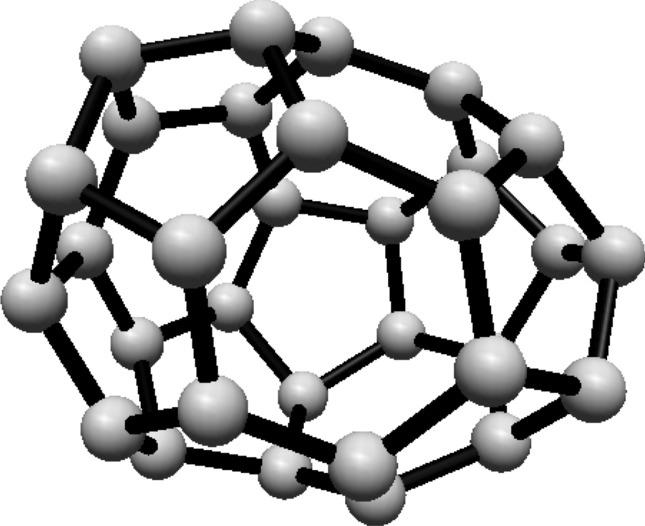
$$ C_{32} $$ Fullerene Structure

 For the fullerene $C_{32}$ see Fig. 8, the graph consists of 13 Hosoya equivalence classes, with cardinalities ranging from 1 to 5.

The normalized proportions are $\lambda _1 = \frac{1}{32}$, $\lambda _2 = \frac{1}{16}$, $\lambda _3 = \frac{3}{32}$, $\lambda _4 = \frac{5}{32}$, and $\lambda _5 = \frac{1}{8}$. Substituting these into Eq. (1):

$$\begin{aligned} H(C_{32})  &   = -\frac{5}{32} \ln \left( \frac{1}{32}\right) - \frac{1}{4} \ln \left( \frac{1}{16}\right) \\  &   \quad - \frac{6}{32} \ln \left( \frac{3}{32}\right) - \frac{10}{32} \ln \left( \frac{5}{32}\right) - \frac{1}{8} \ln \left( \frac{1}{8}\right) \end{aligned}$$

Simplifying:

$$\begin{aligned} H(C_{32})  &   = \frac{5}{32} \ln (32) + \frac{1}{8} \ln (16) - \frac{6}{32} \ln (3) + \frac{6}{32} \ln (32) \\  &   \quad - \frac{10}{32} \ln (5) - \frac{10}{32} \ln (32) + \frac{1}{8} \ln (8) \end{aligned}$$

This simplifies further to:

$$\begin{aligned} H(C_{32})  &   = \frac{37}{16} \ln (2) - \frac{3}{16} \ln (3) \\  &   \quad + \frac{7}{8} \ln (4) - \frac{5}{16} \ln (5) \end{aligned}$$

### Hosoya entropy of fullerene $H(C_{34})$

**Fig. 9:**
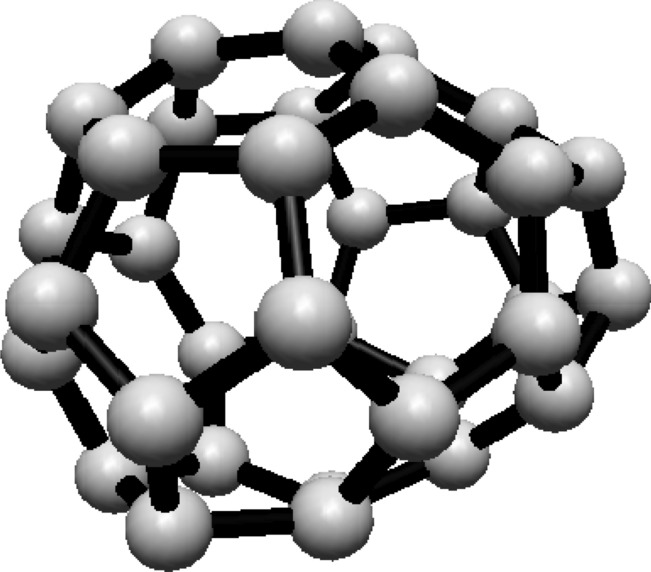
$$ C_{34} $$ Fullerene Structure

For the fullerene $C_{34}$ see Fig. 9, the graph consists of 10 Hosoya equivalence classes, with cardinalities 2, 4, and 6.

The normalized proportions are $\lambda _1 = \frac{1}{17}$, $\lambda _2 = \frac{2}{17}$, and $\lambda _3 = \frac{3}{17}$. Substituting these into Eq. (1):

$$\begin{aligned} H(C_{34})  &   = -\frac{4}{17} \ln \left( \frac{1}{17}\right) - \frac{10}{17} \ln \left( \frac{2}{17}\right) \\  &   \quad - \frac{3}{17} \ln \left( \frac{3}{17}\right) \end{aligned}$$

Simplifying:

$$\begin{aligned} H(C_{34}) = \ln (17) - \frac{10}{17} \ln (2) - \frac{3}{17} \ln (3) \end{aligned}$$

#### Hosoya entropy of fullerene $H(C_{36})$

**Fig. 10:**
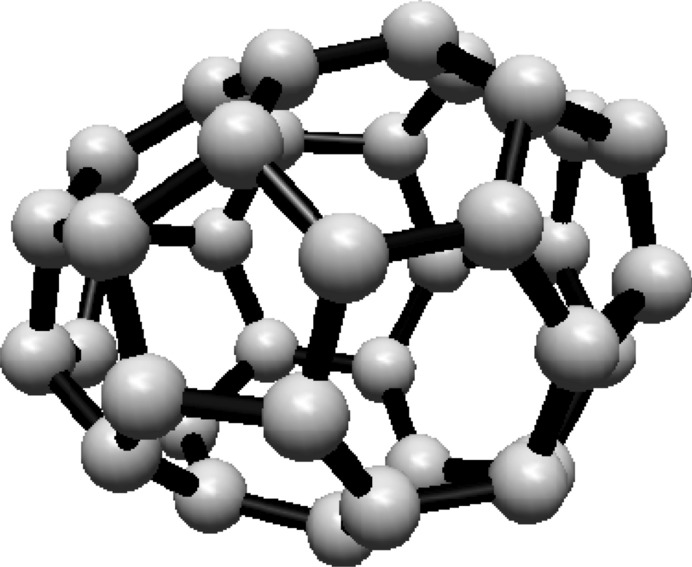
$$ C_{36} $$ Fullerene Structure

 For the fullerene $C_{36}$ see Fig. 10, the graph consists of 12 Hosoya equivalence classes, with cardinalities 2, 4, and 8.

The normalized proportions are $\lambda _1 = \frac{1}{18}$, $\lambda _2 = \frac{1}{9}$, and $\lambda _3 = \frac{2}{9}$. Substituting these into Eq. (1):

$$\begin{aligned} H(C_{36})  &   = -\frac{4}{9} \ln \left( \frac{1}{18}\right) - \frac{1}{3} \ln \left( \frac{1}{9}\right) \\  &   \quad - \frac{2}{9} \ln \left( \frac{2}{9}\right) \end{aligned}$$

Simplifying:

$$\begin{aligned} H(C_{36}) = \ln (9) - \frac{4}{9} \ln (2) \end{aligned}$$

Thus, the Hosoya entropy of $C_{36}$ is:

$$\begin{aligned} H(C_{36}) = \ln (9) - \frac{2}{9} \ln (2) \end{aligned}$$

#### Hosoya entropy of fullerene $H(C_{38})$

**Fig. 11:**
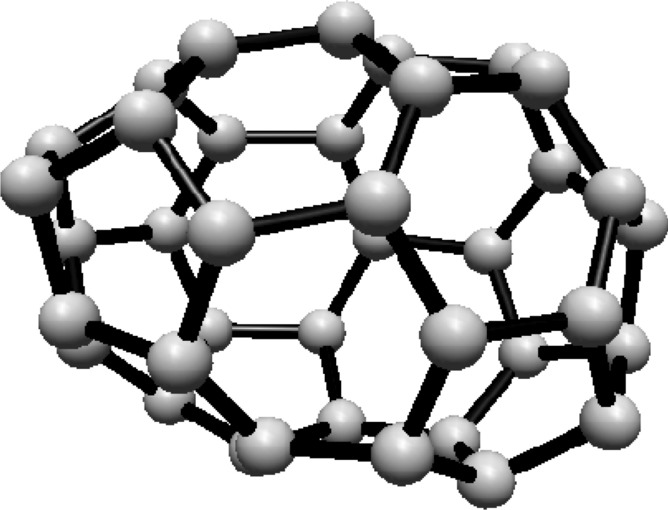
$$ C_{38} $$ Fullerene Structure

 For the fullerene $C_{38}$ see Fig. 11, the graph consists of 15 Hosoya equivalence classes, with cardinalities 2 and 4.

The normalized proportions are $\lambda _1 = \frac{1}{9}$ and $\lambda _2 = \frac{2}{9}$. Substituting these into Eq. (1):

$$\begin{aligned} H(C_{38})  &   = -\frac{11}{19} \ln \left( \frac{1}{19}\right) \\  &   \quad - \frac{8}{19} \ln \left( \frac{2}{19}\right) \end{aligned}$$

Simplifying:

$$\begin{aligned} H(C_{38}) = \ln (19) - \frac{8}{19} \ln (2) \end{aligned}$$

#### Hosoya entropy of fullerene $H(C_{40})$

**Fig. 12:**
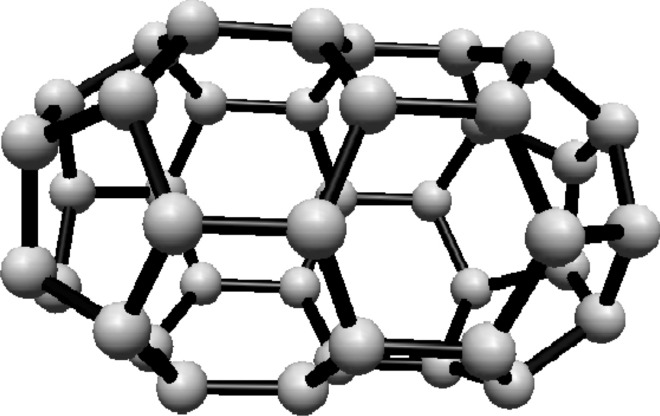
$$ C_{40} $$ Fullerene Structure

 For the fullerene $C_{40}$ see Fig. 12, the graph consists of four Hosoya equivalence classes, each with 10 vertices.

The normalized proportion is $\lambda _1 = \frac{1}{4}$. Substituting this into Eq. (1):

$$\begin{aligned} H(C_{40}) = -\ln \left( \frac{1}{4}\right) \end{aligned}$$

Simplifying:

$$\begin{aligned} H(C_{40}) = \ln (4) \end{aligned}$$

Thus, the Hosoya entropy of $C_{40}$ is $H(C_{40}) = \ln (4)$.

### Hosoya entropy of fullerene $H(C_{42})$

**Fig. 13:**
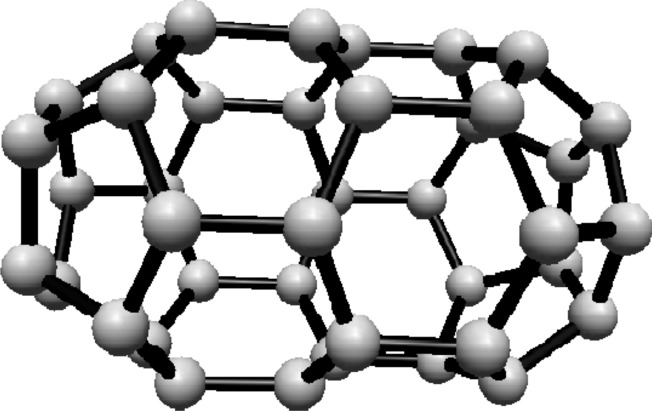
$$ C_{42} $$ Fullerene Structure

For the fullerene $C_{42}$ see Fig. 13, the graph consists of 19 Hosoya equivalence classes. The first 17 classes each contain 2 vertices, and the remaining 2 classes contain 4 vertices.

The normalized proportions are $\lambda _1 = \frac{1}{21}$ and $\lambda _2 = \frac{2}{21}$. Substituting these into Eq. (1):

$$\begin{aligned} H(C_{42})  &   = -\frac{17}{21} \ln \left( \frac{1}{21}\right) \\  &   \quad - \frac{4}{21} \ln \left( \frac{2}{21}\right) \end{aligned}$$

Simplifying:

$$\begin{aligned} H(C_{42}) = \ln (21) - \frac{4}{21} \ln (2) \end{aligned}$$

Thus, the Hosoya entropy of $C_{42}$ is $H(C_{42}) = \ln (21) - \frac{4}{21} \ln (2)$.

### Hosoya entropy of fullerene $H(C_{44})$

**Fig. 14:**
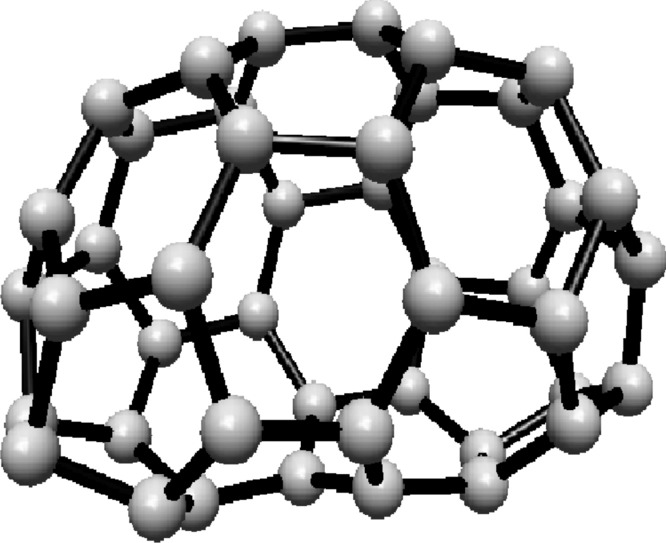
$$C_{44}$$ Fullerene Structure

For the fullerene $C_{44}$ see Fig. 14, the graph consists of 18 Hosoya equivalence classes. The first 14 classes each contain 2 vertices, and the remaining 4 classes each contain 4 vertices.

The normalized proportions are $\lambda _1 = \frac{1}{22}$ and $\lambda _2 = \frac{1}{11}$. Substituting these into Eq. (1):

$$\begin{aligned} H(C_{44})  &   = -\frac{7}{11} \ln \left( \frac{1}{22}\right) \\  &   \quad - \frac{4}{11} \ln \left( \frac{1}{11}\right) \end{aligned}$$

Simplifying:

$$\begin{aligned} H(C_{44}) = \ln (11) + \frac{7}{11} \ln (2) \end{aligned}$$

Thus, the Hosoya entropy of $C_{44}$ is $H(C_{44}) = \ln (11) + \frac{7}{11} \ln (2)$.

### Hosoya entropy of fullerene $H(C_{46})$

**Fig. 15:**
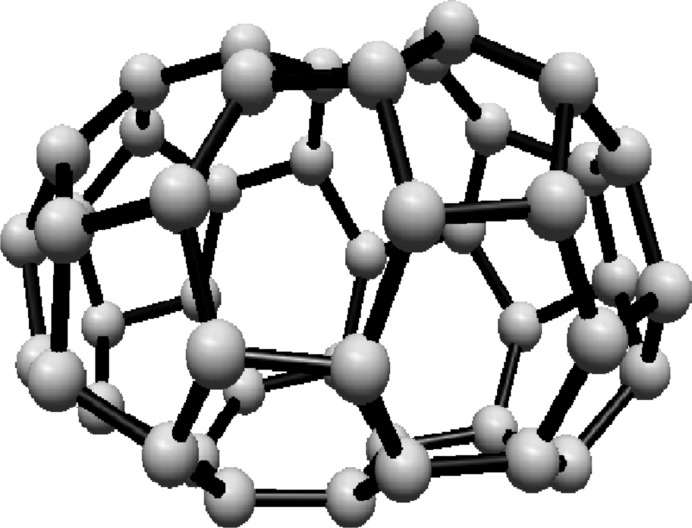
$$ C_{46} $$ Fullerene Structure

For the fullerene $C_{46}$ see Fig. 15, the graph consists of 19 Hosoya equivalence classes. The first 15 classes each contain 2 vertices, and the remaining 4 classes each contain 4 vertices.

The normalized proportions are $\lambda _1 = \frac{1}{23}$ and $\lambda _2 = \frac{2}{23}$. Substituting these into Eq. (1):

$$\begin{aligned} H(C_{46})  &   = -\frac{15}{23} \ln \left( \frac{1}{23}\right) \\  &   \quad - \frac{8}{23} \ln \left( \frac{2}{23}\right) \end{aligned}$$

Simplifying:

$$\begin{aligned} H(C_{46}) = \ln (23) - \frac{8}{23} \ln (2) \end{aligned}$$

Thus, the Hosoya entropy of $C_{46}$ is $H(C_{46}) = \ln (23) - \frac{8}{23} \ln (2)$.

### Hosoya entropy of fullerene $H(C_{48})$

**Fig. 16:**
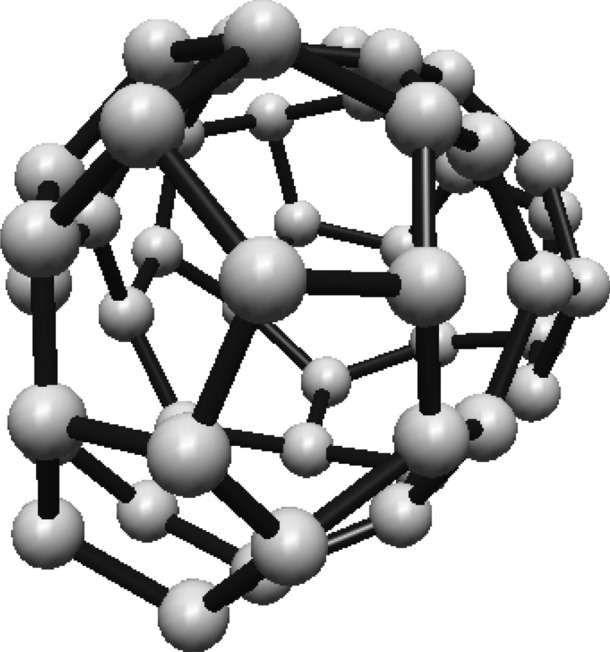
$$ C_{48} $$ Fullerene Structure

For the fullerene $C_{48}$ see Fig. 16, the graph consists of 22 Hosoya equivalence classes. The first 20 classes each contain 2 vertices, and the remaining 2 classes contain 4 vertices.

The normalized proportions are $\lambda _1 = \frac{1}{24}$ and $\lambda _2 = \frac{1}{12}$. Substituting these into Eq. (1):

$$\begin{aligned} H(C_{48}) = -\frac{5}{6} \ln \left( \frac{1}{24}\right) - \frac{1}{6} \ln \left( \frac{1}{12}\right) \end{aligned}$$

Simplifying:

$$\begin{aligned} H(C_{48}) = \ln (12) - \frac{5}{6} \ln (2) \end{aligned}$$

Thus, the Hosoya entropy of $C_{48}$ is $H(C_{48}) = \ln (12) - \frac{5}{6} \ln (2)$.

### Hosoya entropy of fullerene $H(C_{50})$

**Fig. 17:**
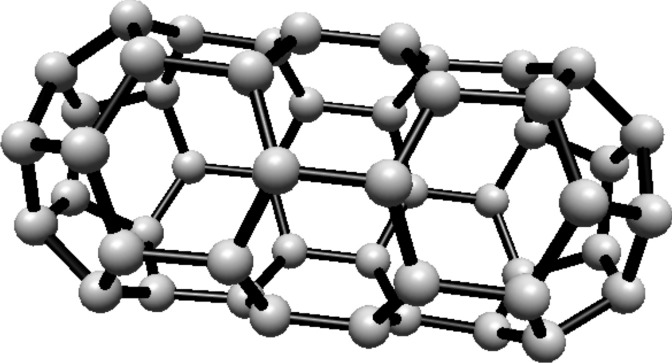
$$ C_{50} $$ Fullerene Structure

For the fullerene $C_{50}$ see Fig. 17, the graph consists of five Hosoya equivalence classes, each containing 10 vertices.

The normalized proportion is $\lambda _1 = \frac{1}{5}$. Substituting this into Eq. (1):

$$\begin{aligned} H(C_{50}) = -\ln \left( \frac{1}{5}\right) \end{aligned}$$

Simplifying:

$$\begin{aligned} H(C_{50}) = \ln (5) \end{aligned}$$

Thus, the Hosoya entropy of $C_{50}$ is $H(C_{50}) = \ln (5)$.

### Hosoya entropy of fullerene $H(C_{52})$

**Fig. 18:**
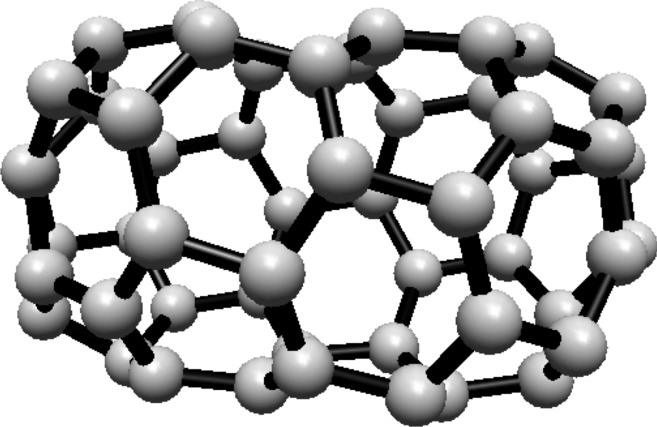
$$ C_{52} $$ Fullerene Structure

For the fullerene $C_{52}$ see Fig. 18, the graph consists of 24 Hosoya equivalence classes. The first 22 classes each contain 2 vertices, and the remaining 2 classes contain 4 vertices.

The normalized proportions are $\lambda _1 = \frac{1}{26}$ and $\lambda _2 = \frac{1}{13}$. Substituting these into Eq. (1):

$$\begin{aligned} H(C_{52}) = -\frac{11}{13} \ln \left( \frac{1}{26}\right) - \frac{2}{13} \ln \left( \frac{1}{13}\right) \end{aligned}$$

Simplifying:

$$\begin{aligned} H(C_{52}) = \ln (13) + \frac{11}{13} \ln (2) \end{aligned}$$

Thus, the Hosoya entropy of $C_{52}$ is $H(C_{52}) = \ln (13) + \frac{11}{13} \ln (2)$.

### Hosoya entropy of fullerene $H(C_{60})$

**Fig. 19:**
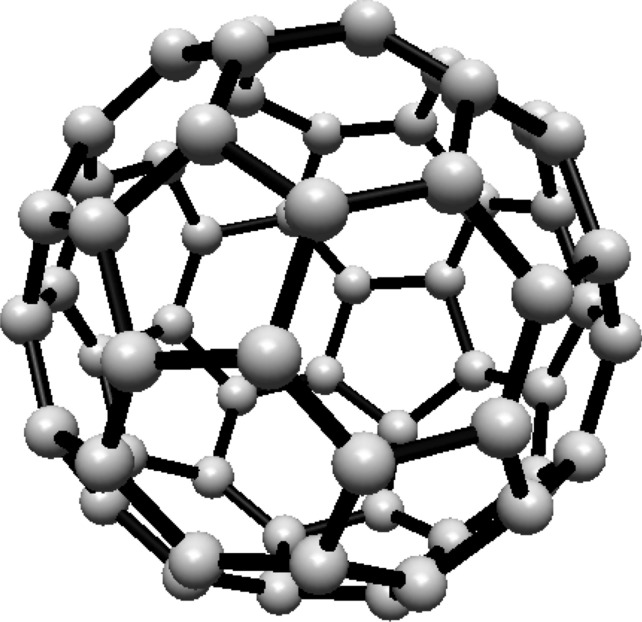
$$ C_{60} $$ Fullerene Structure

For the fullerene $C_{60}$ see Fig. 19, the graph consists of one Hosoya equivalence class containing all 60 vertices.

Since all vertices belong to one class, the normalized proportion is $\lambda _1 = 1$. Substituting this into Eq. (1):

$$\begin{aligned} H(C_{60}) = -1 \cdot \ln (1) = 0 \end{aligned}$$

Thus, the Hosoya entropy of $C_{60}$ is $H(C_{60}) = 0$.

### Hosoya entropy of fullerene $H(C_{70})$

**Fig. 20:**
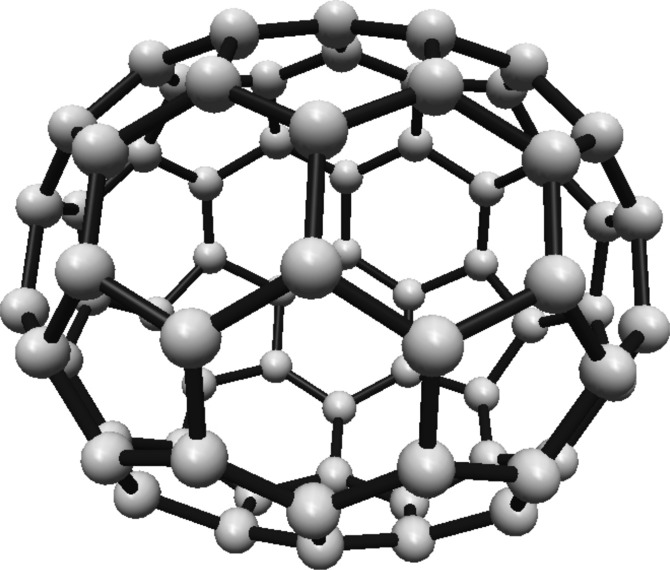
$$ C_{70} $$ Fullerene Structure

For the fullerene $C_{70}$ see Fig. 20, the graph consists of five Hosoya equivalence classes. The first three classes each contain 10 vertices, and the remaining two classes each contain 20 vertices.

The normalized proportions are $\lambda _1 = \frac{1}{7}$ and $\lambda _2 = \frac{2}{7}$. Substituting these into Eq. (1):

$$\begin{aligned} H(C_{70})  &   = -\frac{3}{7} \ln \left( \frac{1}{7}\right) \\  &   \quad - \frac{4}{7} \ln \left( \frac{2}{7}\right) \end{aligned}$$

Simplifying:

$$\begin{aligned} H(C_{70}) = \ln (7) - \frac{4}{7} \ln (2) \end{aligned}$$

Thus, the Hosoya entropy of $C_{70}$ is $H(C_{70}) = \ln (7) - \frac{4}{7} \ln (2)$.

### Hosoya entropy of fullerene $H(C_{72})$

**Fig. 21:**
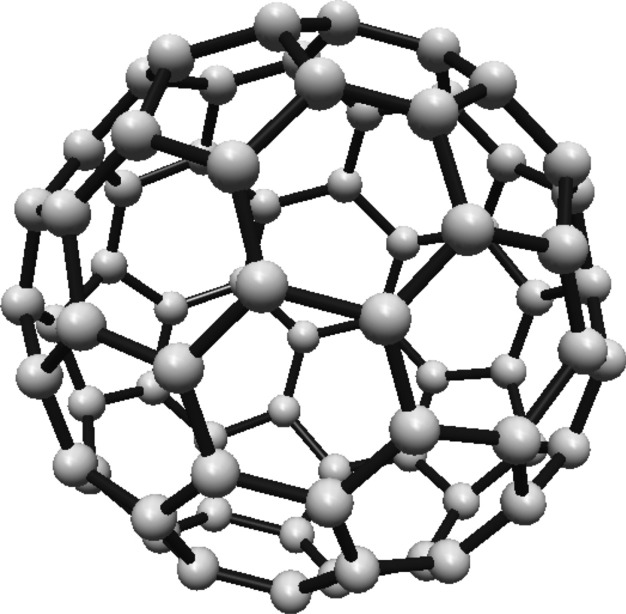
$$ C_{72} $$ Fullerene Structure

For the fullerene $C_{72}$ see Fig. 21, the graph consists of four Hosoya equivalence classes. The first two classes contain 12 vertices each, and the remaining two classes contain 24 vertices each.

The normalized proportions are $\lambda _1 = \frac{1}{6}$ and $\lambda _2 = \frac{1}{3}$. Substituting these into Eq. (1):

$$\begin{aligned} H(C_{72})  &   = -\frac{1}{3} \ln \left( \frac{1}{6}\right) \\  &   \quad - \frac{2}{3} \ln \left( \frac{1}{3}\right) \end{aligned}$$

Simplifying:

$$\begin{aligned} H(C_{72}) = \ln (3) + \frac{1}{3} \ln (2) \end{aligned}$$

Thus, the Hosoya entropy of $C_{72}$ is $H(C_{72}) = \ln (3) + \frac{1}{3} \ln (2)$.

### Hosoya entropy of fullerene $H(C_{74})$

**Fig. 22:**
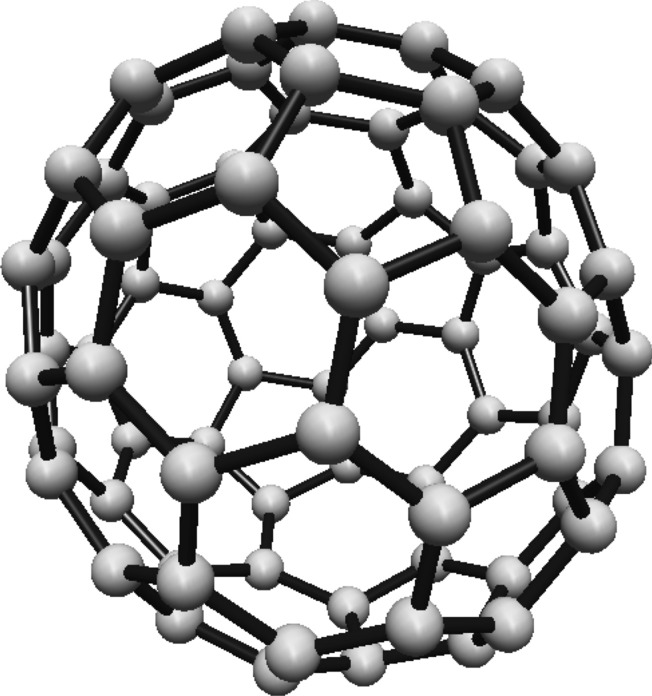
$$ C_{74} $$ Fullerene Structure

For the fullerene $C_{74}$ see Fig. 22, the graph consists of 8 Hosoya equivalence classes. The first class contains 2 vertices, the second group of four classes each contain 6 vertices, and the remaining four classes each contain 12 vertices.

The normalized proportions are $\lambda _1 = \frac{1}{37}$, $\lambda _2 = \frac{3}{37}$, and $\lambda _3 = \frac{6}{37}$. Substituting these into Eq. (1):

$$\begin{aligned} H(C_{74})  &   = -\frac{1}{37} \ln \left( \frac{1}{37}\right) \\  &   \quad - \frac{12}{37} \ln \left( \frac{3}{37}\right) - \frac{24}{37} \ln \left( \frac{6}{37}\right) \end{aligned}$$

Simplifying:

$$\begin{aligned} H(C_{74}) = \ln (37) - \frac{36}{37} \ln (3) - \frac{24}{37} \ln (2) \end{aligned}$$

Thus, the Hosoya entropy of $C_{74}$ is $H(C_{74}) = \ln (37) - \frac{36}{37} \ln (3) - \frac{24}{37} \ln (2)$.

### Hosoya entropy of fullerene $H(C_{76})$

**Fig. 23:**
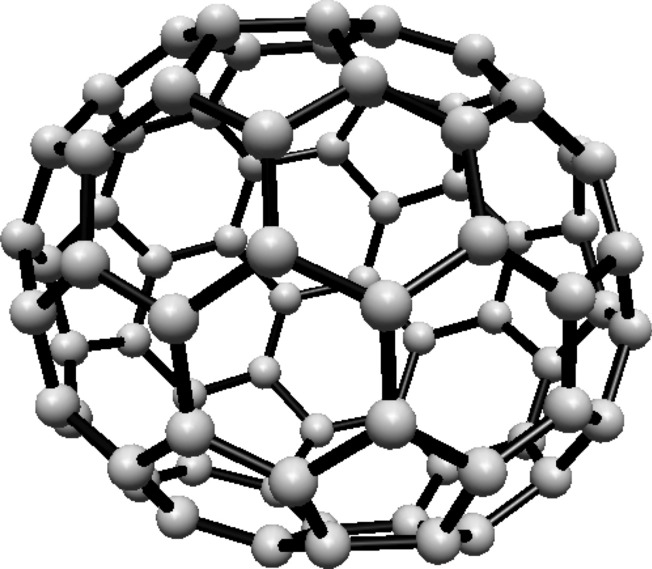
$$ C_{76} $$ Fullerene Structure

For the fullerene $C_{76}$ see Fig. 23, the graph consists of four Hosoya equivalence classes. The first class contains 4 vertices, the second two classes each contain 12 vertices, and the remaining two classes each contain 24 vertices.

The normalized proportions are $\lambda _1 = \frac{1}{19}$, $\lambda _2 = \frac{3}{19}$, and $\lambda _3 = \frac{6}{19}$. Substituting these into Eq. (1):

$$\begin{aligned} H(C_{76})  &   = -\frac{1}{19} \ln \left( \frac{1}{19}\right) - \frac{6}{19} \ln \left( \frac{3}{19}\right) \\  &   \quad - \frac{12}{19} \ln \left( \frac{6}{19}\right) \end{aligned}$$

Simplifying:

$$\begin{aligned} H(C_{76}) = \ln (19) - \frac{12}{19} \ln (3) - \frac{12}{19} \ln (2) \end{aligned}$$

Thus, the Hosoya entropy of $C_{76}$ is $H(C_{76}) = \ln (19) - \frac{12}{19} \ln (3) - \frac{12}{19} \ln (2)$.

### Hosoya entropy of fullerene $H(C_{78})$

**Fig. 24:**
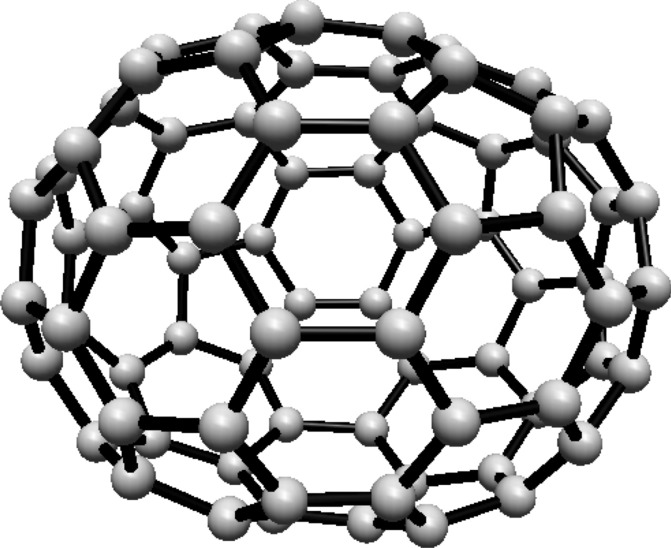
$$ C_{78} $$ Fullerene Structure

For the fullerene $C_{78}$ see Fig. 24, the graph consists of 13 Hosoya equivalence classes, with each class containing 6 vertices.

The normalized proportion is $\lambda _1 = \frac{1}{13}$. Substituting this into Eq. (1):

$$\begin{aligned} H(C_{78}) = - \frac{1}{13} \ln \left( \frac{1}{13}\right) \end{aligned}$$

Simplifying:

$$\begin{aligned} H(C_{78}) = \ln (13) \end{aligned}$$

Thus, the Hosoya entropy of $C_{78}$ is $H(C_{78}) = \ln (13)$.

### Hosoya entropy of fullerene $H(C_{80})$

**Fig. 25:**
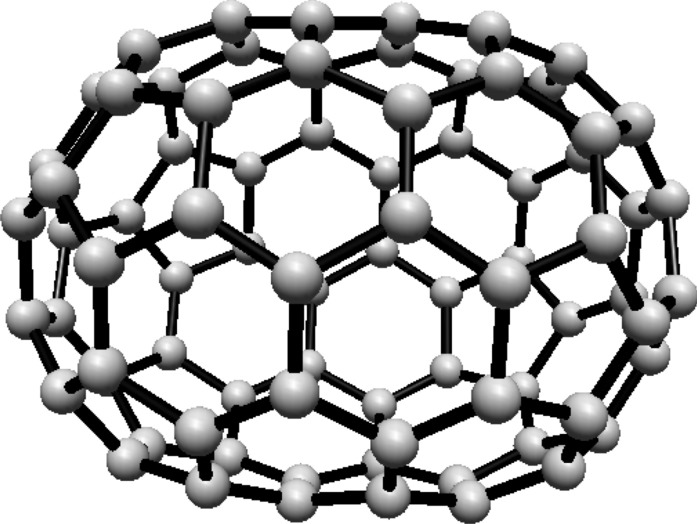
$$ C_{80} $$ Fullerene Structure

For the fullerene $C_{80}$ see Fig. 25, the graph consists of five Hosoya equivalence classes. The first two classes contain 10 vertices each, and the remaining three classes contain 20 vertices each.

The normalized proportions are $\lambda _1 = \frac{1}{8}$ and $\lambda _2 = \frac{1}{4}$. Substituting these into Eq. (1):

$$\begin{aligned} H(C_{80})  &   = -\frac{1}{4} \ln \left( \frac{1}{8}\right) \\  &   \quad - \frac{3}{4} \ln \left( \frac{1}{4}\right) \end{aligned}$$

Simplifying:

$$\begin{aligned} H(C_{80}) = \frac{1}{4} \ln (8) + \frac{3}{4} \ln (4) \end{aligned}$$

Thus, the Hosoya entropy of $C_{80}$ is:

$$\begin{aligned} H(C_{80}) = \frac{9}{4} \ln (2) \end{aligned}$$

### Hosoya entropy of fullerene $H(C_{82})$

**Fig. 26:**
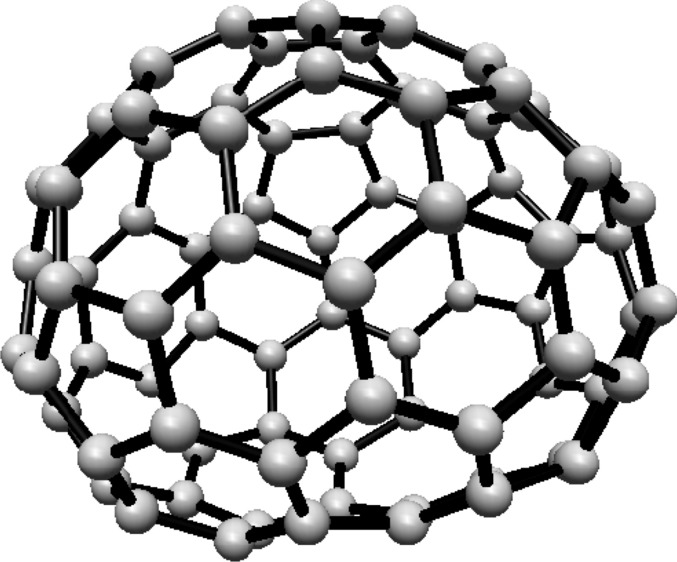
$$ C_{82} $$ Fullerene Structure

For the fullerene $C_{82}$ see Fig. 26, the graph consists of 24 Hosoya equivalence classes. These classes have varying cardinalities of 1, 2, 4, 6, and 7.

The normalized proportions are $\lambda _1 = \frac{1}{82}$, $\lambda _2 = \frac{1}{41}$, $\lambda _3 = \frac{2}{41}$, $\lambda _4 = \frac{3}{41}$, and $\lambda _5 = \frac{7}{82}$. Substituting these into Eq. (1):

$$\begin{aligned} H(C_{82})  &   = -\frac{1}{82} \ln \left( \frac{1}{82}\right) - \frac{14}{41} \ln \left( \frac{1}{41}\right) \\  &   \quad - \frac{8}{41} \ln \left( \frac{1}{41}\right) - \frac{15}{41} \ln \left( \frac{3}{41}\right) \\  &   \quad - \frac{7}{82} \ln \left( \frac{7}{82}\right) \end{aligned}$$

Simplifying:

$$\begin{aligned} H(C_{82}) = \ln (41) + \frac{4}{41} \ln (2) - \frac{15}{41} \ln (3) - \frac{7}{82} \ln (7) \end{aligned}$$

### Hosoya entropy of fullerene $H(C_{84})$

**Fig. 27:**
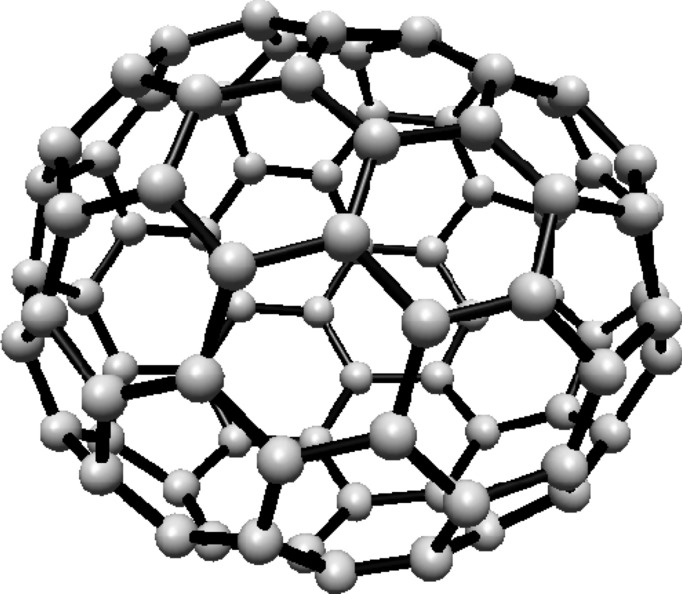
$$ C_{84} $$ Fullerene Structure

For the fullerene $C_{84}$ see Fig. 27, the graph consists of 22 Hosoya equivalence classes with varying cardinalities of 1, 3, and 4.

The normalized proportions are $\lambda _1 = \frac{1}{80}$, $\lambda _2 = \frac{3}{80}$, and $\lambda _3 = \frac{1}{20}$. Substituting these into Eq. (1):

$$\begin{aligned} H(C_{84})  &   = -\frac{1}{80} \ln \left( \frac{1}{80}\right) \\  &   \quad - \frac{3}{80} \ln \left( \frac{3}{80}\right) - \ln \left( \frac{1}{20}\right) \end{aligned}$$

Simplifying:

$$\begin{aligned} H(C_{84}) = \ln (20) - \frac{3}{80} \ln (3) + \frac{1}{20} \ln (4) \end{aligned}$$

### Hosoya entropy of fullerene $H(C_{86})$

**Fig. 28:**
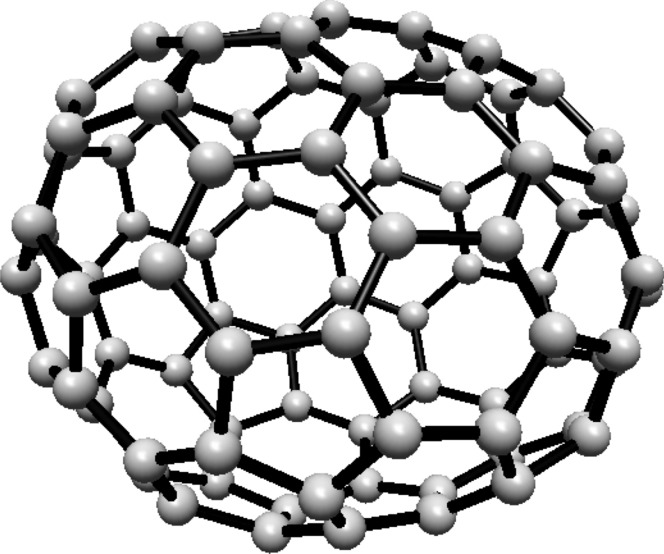
$$ C_{86} $$ Fullerene Structure

For the fullerene $C_{86}$ see Fig. 28, the graph consists of 58 Hosoya equivalence classes with cardinalities of 1, 2, 3, and 4.

The normalized proportions are $\lambda _1 = \frac{1}{86}$, $\lambda _2 = \frac{1}{43}$, $\lambda _3 = \frac{3}{86}$, and $\lambda _4 = \frac{2}{43}$. Substituting these into Eq. (1):

$$\begin{aligned} H(C_{86})  &   = -\frac{37}{86} \ln \left( \frac{1}{86}\right) - \frac{16}{43} \ln \left( \frac{1}{43}\right) \\  &   \quad - \frac{9}{86} \ln \left( \frac{3}{86}\right) - \frac{4}{43} \ln \left( \frac{2}{43}\right) \end{aligned}$$

Simplifying:

$$\begin{aligned} H(C_{86}) = \ln (43) + \frac{19}{43} \ln (2) - \frac{9}{86} \ln (3) \end{aligned}$$

### Hosoya entropy of fullerene $H(C_{90})$

**Fig. 29:**
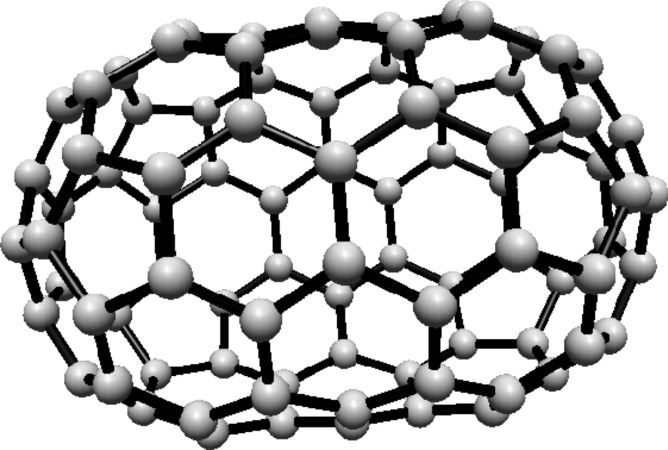
$$ C_{90} $$ Fullerene Structure

For the fullerene $C_{90}$ see Fig. 29, the graph consists of six Hosoya equivalence classes. The first three classes contain 10 vertices each, and the remaining three classes contain 20 vertices each.

The normalized proportions are $\lambda _1 = \frac{1}{9}$ and $\lambda _2 = \frac{2}{9}$. Substituting these into Eq. (1):

$$\begin{aligned} H(C_{90})  &   = -\frac{1}{3} \ln \left( \frac{1}{9}\right) \\  &   \quad - \frac{2}{3} \ln \left( \frac{2}{9}\right) \end{aligned}$$

Simplifying:

$$\begin{aligned} H(C_{90}) = \ln (9) - \frac{2}{3} \ln (2) \end{aligned}$$

### Hosoya entropy of fullerene $H(C_{92})$

**Fig. 30:**
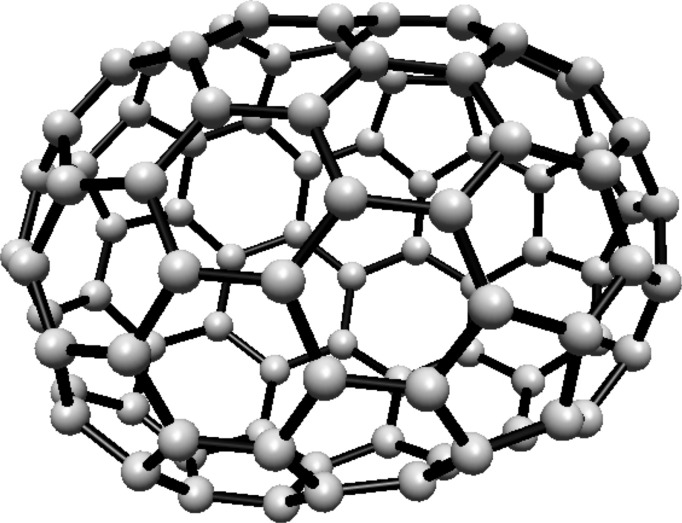
$$ C_{92} $$ Fullerene Structure

For the fullerene $C_{92}$see Fig. 30, the graph consists of 22 Hosoya equivalence classes. The first class contains 8 vertices, and the remaining 21 classes each contain 4 vertices.

The normalized proportions are $\lambda _1 = \frac{1}{23}$ and $\lambda _2 = \frac{2}{23}$. Substituting these into Eq. (1):

$$\begin{aligned} H(C_{92})  &   = -\frac{21}{23} \ln \left( \frac{1}{23}\right) \\  &   \quad - \frac{2}{23} \ln \left( \frac{2}{23}\right) \end{aligned}$$

Simplifying:

$$\begin{aligned} H(C_{92}) = \ln (23) - \frac{2}{23} \ln (2) \end{aligned}$$

Thus, the Hosoya entropy of $C_{92}$ is $H(C_{92}) = \ln (23) - \frac{2}{23} \ln (2)$.

### Hosoya entropy of fullerene $H(C_{94})$

**Fig. 31:**
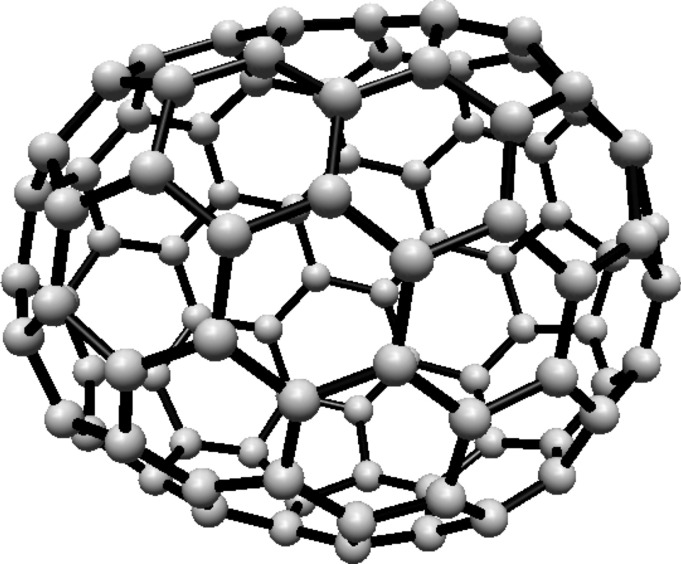
$$ C_{94} $$ Fullerene Structure

For the fullerene $C_{94}$see Fig. 31, the graph consists of 37 Hosoya equivalence classes. These classes have varying cardinalities of 1, 2, 3, 4, and 6.

The normalized proportions are $\lambda _1 = \frac{1}{94}$, $\lambda _2 = \frac{1}{47}$, $\lambda _3 = \frac{3}{94}$, $\lambda _4 = \frac{2}{47}$, and $\lambda _5 = \frac{3}{47}$. Substituting these into Eq. (1):

$$\begin{aligned} H(C_{94})  &   = -\frac{1}{94} \ln \left( \frac{1}{94}\right) - \frac{28}{47} \ln \left( \frac{1}{47}\right) \\  &   \quad - \frac{3}{94} \ln \left( \frac{3}{94}\right) - \frac{14}{47} \ln \left( \frac{2}{47}\right) \\  &   \quad - \frac{3}{47} \ln \left( \frac{3}{47}\right) \end{aligned}$$

Simplifying:

$$\begin{aligned} H(C_{94}) = \ln (47) - \frac{9}{94} \ln (3) - \frac{12}{47} \ln (2) \end{aligned}$$

Thus, the Hosoya entropy of $C_{94}$ is $H(C_{94}) = \ln (47) - \frac{9}{94} \ln (3) - \frac{12}{47} \ln (2)$.

### Hosoya entropy of fullerene $H(C_{96})$

**Fig. 32:**
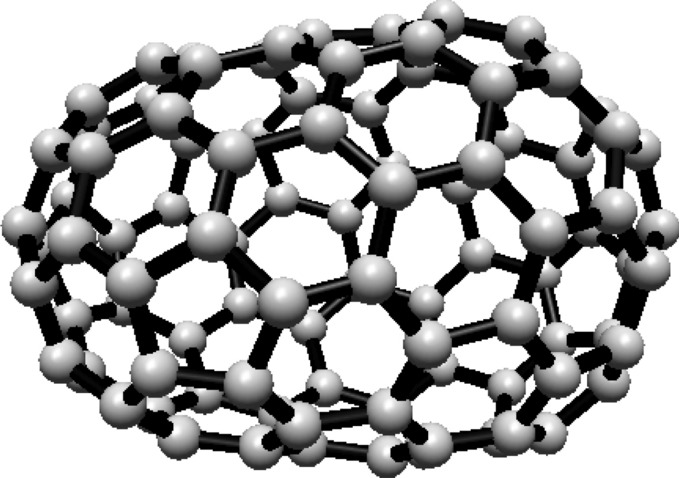
$$ C_{96} $$ Fullerene Structure

For the fullerene $C_{96}$see Fig. 32, the graph consists of 26 Hosoya equivalence classes. These classes have varying cardinalities of 1, 2, 3, and 4.

The normalized proportions are $\lambda _1 = \frac{1}{48}$, $\lambda _2 = \frac{1}{24}$, $\lambda _3 = \frac{1}{48}$, and $\lambda _4 = \frac{5}{24}$. Substituting these into Eq. (1):

$$\begin{aligned} H(C_{96})  &   = -\frac{1}{48} \ln \left( \frac{1}{48}\right) - \frac{1}{12} \ln \left( \frac{1}{24}\right) \\  &   \quad - \frac{3}{48} \ln \left( \frac{1}{48}\right) - \frac{5}{6} \ln \left( \frac{5}{24}\right) \end{aligned}$$

Simplifying:

$$\begin{aligned} H(C_{96}) = \ln (24) - \frac{5}{6} \ln (5) + \frac{1}{12} \ln (2) \end{aligned}$$

Thus, the Hosoya entropy of $C_{96}$ is $H(C_{96}) = \ln (24) - \frac{5}{6} \ln (5) + \frac{1}{12} \ln (2)$.

### Hosoya entropy of fullerene $H(C_{98})$

**Fig. 33:**
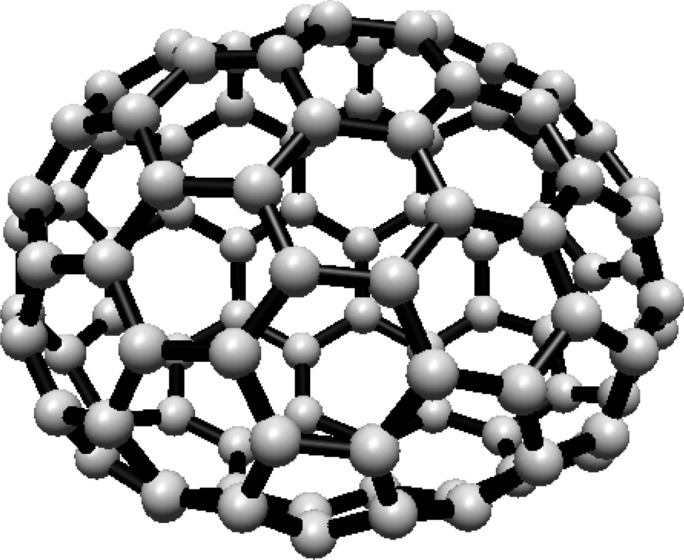
$$ C_{98} $$ Fullerene Structure

For the fullerene $C_{98}$see Fig. 33, the graph consists of 46 Hosoya equivalence classes. The first 43 classes contain 2 vertices each, and the remaining 3 classes contain 4 vertices each.

The normalized proportions are $\lambda _1 = \frac{1}{49}$ and $\lambda _2 = \frac{2}{49}$. Substituting these into Eq. (1):

$$\begin{aligned} H(C_{98}) = -\frac{43}{49} \ln \left( \frac{1}{49}\right) - \frac{6}{49} \ln \left( \frac{2}{49}\right) \end{aligned}$$

Simplifying:

$$\begin{aligned} H(C_{98}) = \ln (49) - \frac{6}{49} \ln (2) \end{aligned}$$

Thus, the Hosoya entropy of $C_{98}$ is $H(C_{98}) = \ln (49) - \frac{6}{49} \ln (2)$.

### Hosoya entropy of fullerene $H(C_{100})$

**Fig. 34:**
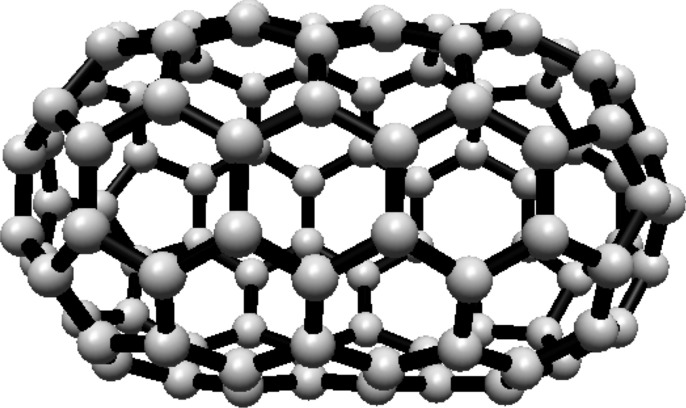
$$ C_{100} $$ Fullerene Structure

For the fullerene $C_{100}$see Fig. 34, the graph consists of six Hosoya equivalence classes. The first two classes each contain 10 vertices, and the remaining four classes each contain 20 vertices.

The normalized proportions are $\lambda _1 = \frac{1}{10}$ and $\lambda _2 = \frac{1}{5}$. Substituting these into Eq. (1):

$$\begin{aligned} H(C_{100}) = -\frac{1}{5} \ln \left( \frac{1}{10}\right) - \frac{4}{5} \ln \left( \frac{1}{5}\right) \end{aligned}$$

Simplifying:

$$\begin{aligned} H(C_{100}) = \ln (5) + \frac{1}{5} \ln (2) \end{aligned}$$

Thus, the Hosoya entropy of $C_{100}$ is $H(C_{100}) = \ln (5) + \frac{1}{5} \ln (2)$. s

## Conclusion

This study examined the relationship between molecular symmetry and Hosoya entropy for various fullerene structures, including $F^s_{3,1},$ $F^s_{4,2},$ and fullerenes ranging from $C_{20}$ to $C_{100}.$ Our results demonstrate that symmetric fullerenes, such as $C_{60}$ and $C_{70},$ exhibit lower Hosoya entropy due to the homogeneity in the distribution of equivalence classes. Conversely, less symmetric fullerenes, such as $C_{26},$ have higher entropy, reflecting their greater structural irregularities. This highlights the utility of Hosoya entropy as a theoretical tool for quantifying molecular complexity.

The Hosoya entropy values calculated in this study provide valuable insights into the structural regularity and intricacy of fullerenes. Larger fullerenes, with their greater number of vertices and edges, tend to exhibit higher entropy due to the increased complexity in their equivalence class distributions. For instance, $C_{20},$ a smaller and more symmetric fullerene, has lower entropy compared to $C_{100},$ which exhibits significantly higher entropy due to its greater structural diversity.

Our analysis emphasizes the critical role of the dispersion in equivalence class distributions in determining the Hosoya entropy of fullerenes. Fullerenes with more evenly distributed equivalence classes, such as $C_{60}$ and $C_{70},$ exhibit lower entropy compared to those with a broader spread of class cardinalities. This indicates that internal symmetry and regularity are essential factors in determining the entropy of fullerene structures.

In short, this present work puts to light Hosoya entropy as the effective measure in describing the extent of structural complexity and stability related to fullerene molecules. Establishing its relevance to both molecular symmetry and diversity, it paves the way for prospective studies. For example, future research might increase the scope further by considering either other families of molecules or studying correlations between Hosoya entropy and other important properties of a molecule.

## Data Availability

All the data used to finding the results is included in the manuscript.
